# Uric Acid-to-Albumin Ratio as a Complementary Biomarker for In-Hospital Risk Stratification in Patients with Pulmonary Hypertension: A Retrospective Cohort Study

**DOI:** 10.3390/jcdd13070295

**Published:** 2026-06-25

**Authors:** Yuanzheng Ye, He Wang, Yongying Lan, Wenqi Pan, Ning Zhang, Tianyou Ling, Yun Xie, Hongzhen Wang, Qiancheng Ma, Chengze Lin, Baopeng Tang, Liqun Wu

**Affiliations:** 1 Department of Cardiovascular Disease Center, The First Affiliated Hospital of Xinjiang Medical University, Urumqi 830054, China; yuanzhengye@xjmu.edu.cn; 2 School of Clinical Medicine, Xinjiang Medical University, Urumqi 830054, China; wh20040909@stu.xjmu.edu.cn (H.W.); 15999298873@stu.xjmu.edu.cn (Q.M.); 3 Department of Cardiology, Shanghai Ruijin Hospital, Shanghai Jiao Tong University School of Medicine, Shanghai 200025, China; lanyongying@sjtu.edu.cn (Y.L.); pwq10859@rjh.com.cn (W.P.); zn11476@rjh.com.cn (N.Z.); lty11498@rjh.com.cn (T.L.); xy11992@rjh.com.cn (Y.X.); whz11452@rjh.com.cn (H.W.); lcz0824@sjtu.edu.cn (C.L.)

**Keywords:** uric acid-to-albumin ratio, pulmonary hypertension, oxidative stress, in-hospital mortality, risk stratification

## Abstract

**Background**: Oxidative stress is pivotal in pulmonary hypertension. The uric acid-to-albumin ratio (UAR) is a readily available composite biomarker reflecting oxidative stress, inflammation and nutritional status. However, its clinical value for short-term risk stratification in PH remains unclear. **Objective**: This study aimed to evaluate the association of UAR with in-hospital mortality, clinically recorded PH severity grades, and selected cardiac structural and functional indicators in hospitalized patients with PH. **Methods**: This single-center retrospective cohort study included 8763 PH patients. Patients were stratified by UAR quartiles. Ordinal logistic regression, multivariable logistic regression, and linear regression were used to assess associations of UAR with clinically recorded PH severity grades, in-hospital mortality, left ventricular ejection fraction (LVEF), and right ventricular internal diameter (RVID). Restricted cubic spline analyses, subgroup analyses, receiver operating characteristic curve analyses, and incremental prediction analyses using C-statistics, net reclassification improvement (NRI), and integrated discrimination improvement (IDI) were also performed. **Results**: In-hospital mortality increased stepwise across UAR quartiles (0.5% vs. 1.0% vs. 1.8% vs. 2.8%, *p* < 0.001). In the fully adjusted model, each 1-unit increase in UAR was associated with higher odds of a more severe clinically recorded PH grade (OR = 1.11, 95% CI: 1.09–1.13, *p* < 0.001) and higher odds of in-hospital mortality (OR = 1.09, 95% CI: 1.04–1.14, *p* < 0.001). Higher UAR was also associated with lower LVEF (β = −0.53, 95% CI: −0.58 to −0.47, *p* < 0.001) and greater RVID (β = 0.18, 95% CI: 0.15–0.22, *p* < 0.001). Adding UAR to a model containing routinely available clinical, laboratory, and echocardiographic variables improved the C-statistic from 0.6922 to 0.7230, with significant improvements in NRI and IDI. **Conclusions**: UAR was independently associated with in-hospital mortality, clinically recorded PH severity, LVEF, and RVID, and provided incremental prognostic information. UAR may serve as a low-cost, routinely available, complementary biomarker for short-term in-hospital risk stratification in patients with PH.

## 1. Introduction

Pulmonary hypertension (PH) is a progressive cardiovascular disorder characterized by increased pulmonary vascular resistance and elevated pulmonary arterial pressure, which can lead to right ventricular remodeling, progressing to right ventricular failure and eventual mortality [1]. Independently of the underlying disease, the development of pulmonary hypertension is associated with clinical deterioration and a substantially increased mortality risk [2]. As such, it is a substantial global health issue that accounts for a considerable health-associated burden worldwide, particularly in countries with ageing populations [3]. This issue is especially pronounced in Asian regions [4]. Oxidative stress, recognized as a key pathogenic mechanism in PH, involves increased production and accumulation of reactive oxygen species (ROS). This process can exacerbate endothelial dysfunction and promote vascular smooth muscle cell proliferation, thereby accelerating disease progression [5]. Additionally, oxidative stress may influence right ventricular remodeling secondary to PH, further raising the risk of clinical deterioration and mortality [6]. Existing studies have provided evidence of heightened oxidative stress in the lungs and pulmonary vasculature across both different clinical classifications of patients with PH (Groups I to V) and relevant PH animal models [6,7].

Serum uric acid (UA), the end product of purine degradation, has been proposed as a biomarker of oxidative stress-related damage [8]. Elevated UA levels or hyperuricemia have been demonstrated to be risk factors for the incidence, progression, and adverse prognosis of various diseases, including cardiovascular diseases, metabolic disorders, diabetes, and renal diseases [8,9,10,11]. Multiple studies have indicated that baseline serum UA concentrations can predict the severity of PH, adverse outcomes, and long-term mortality [12,13,14]. Serum albumin (ALB) serves as a biomarker of nutritional and inflammatory status and also exhibits antioxidant properties in redox balance. One study revealed that lower ALB levels are associated with higher mortality rates in patients with PH [15]. Recently, the uric acid-to-albumin ratio (UAR) has emerged as a readily available composite biomarker that integrates information related to oxidative stress, inflammation, nutritional status, and renal-metabolic burden in cardiovascular diseases [16]. Evidence suggests that UAR independently predicts the severity of coronary artery disease, adverse prognosis in heart failure patients and post-cardiac surgery outcomes, as well as all-cause and cardiovascular mortality in patients with diabetes [17,18,19,20,21]. Moreover, one study demonstrated that UAR exhibits superior predictive performance compared to either UA or albumin alone [22].

However, the clinical value of UAR in patients with PH, particularly for short-term in-hospital risk stratification, remains unclear. Given that UAR integrates information related to oxidative stress, inflammation, nutritional status, and renal-metabolic burden, it may provide complementary prognostic information in hospitalized patients with PH. Therefore, we conducted a retrospective cohort study to evaluate the association between UAR and in-hospital mortality in patients with clinically diagnosed PH. We also examined whether UAR was associated with clinically recorded PH severity grades and selected indicators related to cardiac functional status, structural remodeling, and cardiac burden, including LVEF and RVID. Furthermore, we assessed whether UAR adds prognostic information beyond currently accepted clinical, laboratory, and imaging markers, aiming to better clarify its clinical value and potential application scenario in patients with PH.

## 2. Methods

### 2.1. Study Design and Population

This single-center retrospective cohort study enrolled 22,533 consecutive patients with pulmonary hypertension who were diagnosed or treated at the First Affiliated Hospital of Xinjiang Medical University between January 2015 and March 2025. The study adhered to the principles of the Declaration of Helsinki and was approved by the Ethics Committee of the First Affiliated Hospital of Xinjiang Medical University (approval no.: K202504-79). Given the retrospective design and use of anonymized patient data, the requirement for informed consent was waived.

**Figure 1:**
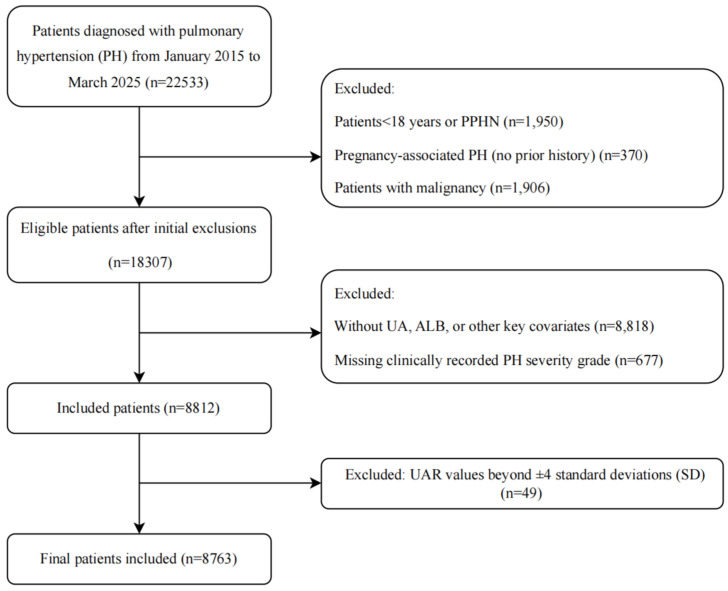
Flow chart of the study population and exclusion criteria. Flowchart illustrating the recruitment and exclusion criteria of the study population. A total of 8763 patients were included in the final analysis.

The diagnosis and clinical identification of pulmonary hypertension (PH) were guided by current ESC/ERS recommendations, in which right-heart catheterization is regarded as the reference standard for definitive hemodynamic diagnosis [7]. However, because this study was based on a real-world retrospective hospitalized cohort, right-heart catheterization data were not available for all patients. Therefore, patients with PH were identified from electronic medical records by integrating available clinical diagnosis records, medical history, echocardiographic findings suggestive of PH, and the treating physicians’ comprehensive clinical assessment during hospitalization. Among 22,533 patients diagnosed with PH, we applied several exclusion criteria: (1) patients under 18 years of age or diagnosed with persistent pulmonary hypertension of the newborn (n = 1950); (2) women with pregnancy-associated PH but no previous history of PH (n = 370); and (3) patients with concomitant malignancies (n = 1906). After these initial exclusions, 18,307 eligible patients remained. We further excluded patients with missing data on UA, ALB, or other key covariates (n = 8818), those without clinically recorded PH severity grades of mild, moderate, or severe PH (n = 677), and those with UAR values beyond ±4 standard deviations (SD) (n = 49). Ultimately, a total of 8763 patients with PH were included in the final analysis (Figure 1).

### 2.2. Data Collection and Definitions

Demographic data and personal medical histories recorded at admission were extracted from the electronic medical records system, including age, sex, race, body mass index (BMI), hypertension, diabetes mellitus, chronic kidney disease (CKD), and connective tissue disease (CTD). Clinically recorded PH severity grades, including mild, moderate, and severe PH, were also extracted from the medical records. These clinically recorded severity categories were generally based on routine clinical assessment, particularly echocardiographic estimates of pulmonary artery pressure and right-heart burden, and similar echocardiography-based mild, moderate, and severe PH classifications have been applied in previous guideline-supported echocardiographic assessments and clinical studies [7,23,24,25,26,27]. Peripheral venous blood samples and other laboratory tests were obtained within 24 h of admission, encompassing measurements of NT-proBNP, white blood cell count, lymphocyte count, neutrophil count, monocyte count, hemoglobin, platelet count, ALT, AST, albumin, globulin, creatinine, urea, uric acid, glucose, total cholesterol, triglycerides, HDL-C, and LDL-C. Echocardiography and blood gas analysis were completed within 48 h of admission. The uric acid-to-albumin ratio (UAR) was calculated as the serum uric acid concentration (μmol/L) divided by the serum albumin level (g/L).

### 2.3. Study Outcome Events

The primary outcome of this study was all-cause in-hospital mortality among patients with PH. In-hospital mortality was defined as death from any cause during hospitalization after baseline assessment. In addition, clinically recorded PH severity grades were evaluated as ordered clinical categories. We also examined the cross-sectional associations of UAR with left ventricular ejection fraction (LVEF) and right ventricular internal diameter (RVID), which were selected as available indicators related to cardiac functional status, structural remodeling, and cardiac burden in patients with PH.

### 2.4. Statistical Analysis

All participants were stratified into quartiles based on UAR levels, and their characteristics were delineated. Categorical variables were shown as numbers (n) with percentages (%). The Kolmogorov–Smirnov test was used to check the normal distribution of continuous variables. Normally distributed data were expressed as means with standard deviations (SD), while skewed data were shown as medians with interquartile ranges (IQR). Continuous variables were compared using one-way analysis of variance or Kruskal–Wallis tests, as appropriate. Categorical variables were compared using chi-square tests or Fisher’s exact tests [28].

Univariate logistic regression analysis was first employed to identify variables associated with in-hospital mortality in patients with PH. Ordinal logistic regression analysis was then performed to evaluate the association between UAR and clinically recorded PH severity grades, with mild, moderate, and severe PH treated as ordered outcome categories. Subsequently, multivariable logistic regression analysis was used to determine the independent association between UAR and in-hospital mortality, whereas multivariable linear regression analysis was employed to assess the cross-sectional associations of UAR with LVEF and RVID.

Before constructing the multivariable models, collinearity between UAR and other variables was examined by calculating the variance inflation factor (VIF), with VIF ≥ 5 considered indicative of significant collinearity. Covariate selection was guided by the following criteria: (1) variables whose inclusion or exclusion changed the effect estimate by more than 10%; (2) variables significantly associated with the outcome in univariate analysis (*p* < 0.05); and (3) variables considered clinically relevant or previously reported in the literature. In the primary multivariable analyses, UAR was analyzed both as a continuous variable and according to quartiles. Sequential models were constructed as follows: Model 1 was adjusted for age, sex, race, and BMI; Model 2 was further adjusted for hypertension, diabetes, CKD, and CTD; Model 3 was further adjusted for NT-proBNP, creatinine, TC, TG, HDL-C, and LDL-C; and Model 4 was further adjusted for CaO_2_, SaO_2_, and PaO_2_.

To explore the dose–response relationships of UAR with in-hospital mortality, LVEF, and RVID, restricted cubic spline (RCS) analyses were performed using the fully adjusted model, with four knots and the median UAR value as the reference point. When a nonlinear association was detected, threshold effect analysis was performed to estimate the most likely inflection point, followed by two-piecewise regression models on either side of the threshold. Subgroup analyses were conducted to evaluate the robustness and potential effect modification of the associations of UAR with in-hospital mortality, LVEF, and RVID across clinically relevant subgroups.

Receiver operating characteristic (ROC) curve analysis was performed to evaluate the predictive performance of different models for in-hospital mortality. Incremental predictive value was assessed by comparing the C-statistic, net reclassification improvement (NRI), and integrated discrimination improvement (IDI) before and after adding UAR to models containing routinely available clinical, laboratory, and echocardiographic variables.

Statistical significance was set at *p* < 0.05 (two-sided). Analysis was performed using R 4.2.2 (http://www.R-project.org; accessed on 20 November 2025; The R Foundation, Vienna, Austria) and the Free Statistics software (version 2.5.1; Beijing FreeClinical Medical Technology Co., Ltd., Beijing, China).

## 3. Results

### 3.1. Baseline Characteristics

A total of 8763 patients with pulmonary hypertension were enrolled, with a mean age of 64.6 ± 15.0 years, 54.7% females, and 57.8% Han Chinese. The mean value of UAR was 9.1 ± 3.7. The baseline characteristics of participants according to UAR quartiles are presented in Table 1. Significant differences among UAR quartiles were observed in most baseline characteristics, including demographics, comorbidities, vital signs, echocardiographic parameters, laboratory data, clinically recorded PH severity grades, and in-hospital mortality. The proportions of male patients, hypertension, diabetes, CKD, moderate-to-severe PH, and LVEF ≤ 40% and 41–49% increased with increasing UAR quartiles. Patients with higher UAR levels tended to have higher levels of UA, WBC, neutrophil count, monocyte count, ALT, AST, creatinine, and urea. Notably, NT-proBNP levels increased progressively from Q1 to Q4 in accordance with rising UAR quartiles. Conversely, ALB, PLT, TC, HDL-C, and SaO_2_ were lower in patients with higher UAR levels. Echocardiographic measurements demonstrated a trend toward increasing RVID and other cardiac chamber dimensions with rising UAR, whereas LVEF decreased from 61.4 ± 5.8% in Q1 to 54.4 ± 11.5% in Q4. The proportion of severe PH also increased from 8.3% in Q1 to 21.2% in Q4. The incidence of in-hospital mortality increased stepwise with increasing UAR quartiles (0.5% vs. 1.0% vs. 1.8% vs. 2.8%, *p* < 0.001). To further describe disease-severity-related clinical heterogeneity, baseline characteristics according to clinically recorded PH severity grades are presented in Table S1.

### 3.2. Factors Associated with In-Hospital Mortality of PH

The results of the univariate regression analyses are presented in Table S2. We found that age, race, monocyte, UA, Urea, ALB, TC, CaO_2_ were correlated with in-hospital mortality in pulmonary hypertension patients (*p* < 0.05).

### 3.3. Association Between UAR and Clinically Recorded PH Severity Grades

To further evaluate the relationship between UAR and clinically recorded PH severity, ordinal logistic regression analyses were performed using mild, moderate, and severe PH as ordered outcome categories. As shown in Table 2, higher UAR was consistently associated with greater odds of being classified into a higher PH severity grade. The association remained significant after sequential adjustment for demographics, comorbidities including CKD and CTD, NT-proBNP, renal function, lipid profiles, and oxygenation parameters. In the fully adjusted model, each 1-unit increase in UAR was still associated with 11% higher odds of a more severe clinically recorded PH grade (OR = 1.11, 95% CI: 1.09 to 1.13, *p* < 0.001).

When UAR was analyzed according to quartiles, a graded association was also observed. Compared with patients in the lowest UAR quartile, those in Q2, Q3, and Q4 had progressively higher odds of being classified into a higher PH severity grade in the fully adjusted model, with ORs of 1.33 (95% CI: 1.22 to 1.45), 1.82 (95% CI: 1.69 to 1.96), and 2.99 (95% CI: 2.81 to 3.19), respectively; all *p* values were <0.001. These findings suggest that elevated UAR is associated with greater clinically recorded PH disease burden.

### 3.4. Associations of UAR with In-Hospital Mortality, LVEF, and RVID

In this study, we constructed four models to analyze the independent associations of UAR with in-hospital mortality and echocardiographic markers of cardiac remodeling/function in patients with PH. The effect estimates and 95% confidence intervals (CIs) are listed in Table 3. When UAR was analyzed as a continuous variable, UAR showed a significantly positive association with the odds of in-hospital mortality. In the unadjusted model, each 1-unit increase in UAR was associated with 13% higher odds of in-hospital mortality (OR = 1.13, 95% CI: 1.09 to 1.18, *p* < 0.001). Models 1–3 all showed consistent statistical significance. In the fully adjusted model (Model 4), each 1-unit increase in UAR remained associated with 9% higher odds of in-hospital mortality (OR = 1.09, 95% CI: 1.04 to 1.14, *p* < 0.001). The results were also significant when UAR was converted from a continuous variable to quartiles. Compared with patients in the lowest UAR quartile, those in Q3 and Q4 had significantly higher odds of in-hospital mortality in the fully adjusted model, with ORs of 3.06 (95% CI: 1.57 to 5.95, *p* = 0.001) and 4.28 (95% CI: 2.20 to 8.35, *p* < 0.001), respectively. These findings indicate that UAR was independently associated with in-hospital mortality in patients with PH.

We also examined the cross-sectional associations of UAR with LVEF and RVID to explore whether UAR was related to echocardiographic markers of cardiac remodeling/function. When analyzed as a continuous variable, UAR demonstrated a significant inverse association with LVEF across both unadjusted and fully adjusted models. In the fully adjusted model, each 1-unit increase in UAR was associated with a 0.53 percentage-point decrease in LVEF (β = −0.53, 95% CI: −0.58 to −0.47, *p* < 0.001). When UAR was analyzed according to quartiles, patients in Q2, Q3, and Q4 had progressively lower LVEF compared with those in Q1, with β coefficients of −0.77 (95% CI: −1.27 to −0.26, *p* = 0.003), −1.94 (95% CI: −2.45 to −1.42, *p* < 0.001), and −4.95 (95% CI: −5.50 to −4.39, *p* < 0.001), respectively, in the fully adjusted model. In the analysis of RVID, UAR showed a significant positive association with RVID when treated as a continuous variable. In the fully adjusted model, each 1-unit increase in UAR was associated with a 0.18 mm increase in RVID (β = 0.18, 95% CI: 0.15 to 0.22, *p* < 0.001). Compared with patients in Q1, those in Q3 and Q4 had significantly larger RVID, with β coefficients of 0.76 mm (95% CI: 0.46 to 1.05, *p* < 0.001) and 1.79 mm (95% CI: 1.48 to 2.11, *p* < 0.001), respectively. Collectively, these results suggest that higher UAR was associated not only with increased in-hospital mortality, but also with greater cardiac remodeling and poorer cardiac functional status, supporting its potential relevance to both short-term prognosis and cardiac burden in hospitalized patients with PH.

**Figure 2:**
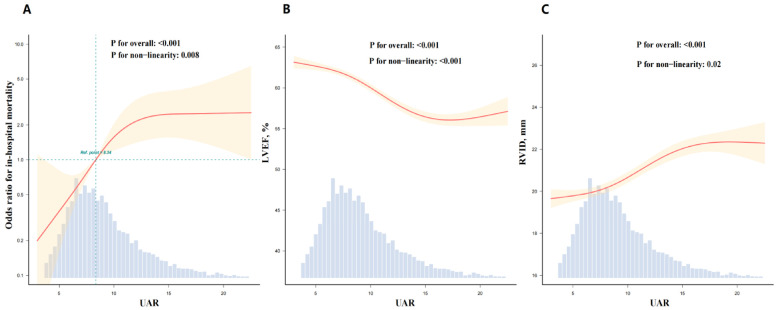
Dose–response associations of UAR with in-hospital mortality, LVEF, and RVID. (**A**) Association between UAR and in-hospital mortality. (**B**) Association between UAR and LVEF. (**C**) Association between UAR and RVID. Curves were fitted using restricted cubic splines in the fully adjusted model, and UAR values were plotted between the 0.5th and 99.5th percentiles. Shaded areas indicate 95% confidence intervals, and histograms show the distribution of UAR. The solid red line represents the fitted RCS curve, the shaded area represents the 95% confidence interval, the blue bars represent the distribution of UAR, and the dashed lines indicate the reference point/level.

To explore the dose–response relationships of UAR with in-hospital mortality, LVEF, and RVID in patients with PH, we performed curve fitting using restricted cubic spline (RCS) models with the median UAR value as the reference point. In the fully adjusted model, significant non-linear associations were observed between UAR and all three outcomes, including in-hospital mortality (*p* for non-linearity = 0.008; Figure 2A), LVEF (*p* for non-linearity <0.001; Figure 2B), and RVID (*p* for non-linearity = 0.020; Figure 2C). Threshold effect analyses were further performed to characterize these non-linear patterns. For in-hospital mortality, the inflection point was identified at UAR = 11.3. Below this threshold, higher UAR was significantly associated with higher odds of in-hospital mortality (OR = 1.298, 95% CI: 1.133 to 1.486, *p* < 0.001), whereas the association was no longer statistically significant above this threshold (*p* = 0.519). For LVEF, the inflection point was identified at UAR = 16.3. When UAR was <16.3, higher UAR was significantly associated with lower LVEF (β = −0.645, 95% CI: −0.714 to −0.576, *p* < 0.001), whereas the association was not statistically significant when UAR was ≥16.3 (*p* = 0.878). For RVID, although the RCS curve suggested a non-linear positive association, the likelihood ratio test did not support a statistically significant threshold effect (*p* = 0.538). Therefore, the RVID result was interpreted as a generally positive association across the observed UAR range rather than a confirmed threshold-dependent relationship. Complete results of the threshold effect analyses are shown in Table S3.

### 3.5. Subgroup Analyses

We further performed subgroup analyses to investigate whether the associations of UAR with in-hospital mortality, LVEF, and RVID in patients with PH were stable across clinically relevant subgroups. In the fully adjusted model, stratified analyses were performed by age (<65 years vs. ≥65 years), sex, BMI (≤25 kg/m^2^, 25–30 kg/m^2^, ≥30 kg/m^2^), hypertension, diabetes, CKD, heart failure with preserved ejection fraction (HFpEF) status, and clinically recorded PH severity grades.

**Figure 3:**
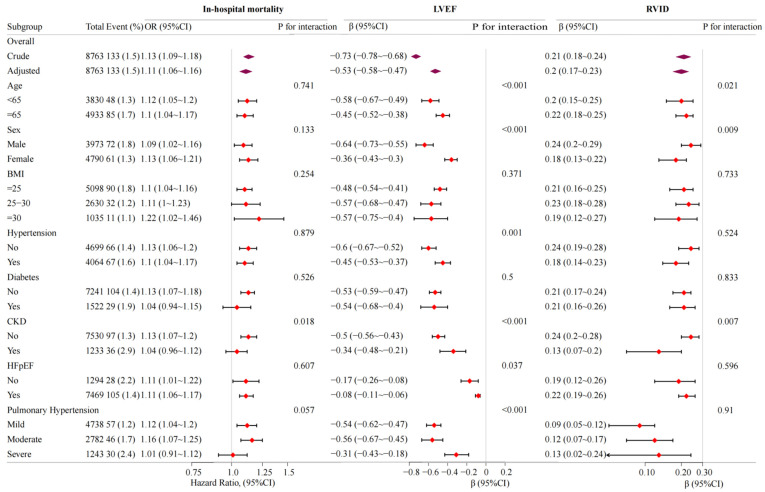
Subgroup analyses of the associations of UAR with in-hospital mortality, LVEF, and RVID. Subgroup analyses were performed according to age, sex, BMI, hypertension, diabetes, CKD, HFpEF status, and clinically recorded PH severity grades. UAR was modeled as a continuous variable per 1-unit increase. ORs and 95% CIs are shown for in-hospital mortality, whereas β coefficients and 95% CIs are shown for LVEF and RVID. *p* values for interaction were calculated to assess effect modification across subgroups.

The forest plot (Figure 3) showed for in-hospital mortality, the significantly positive association with UAR was generally consistent across most subgroups. The consistency observed further supports the robustness and reliability of these conclusions. Notably, the significant association was similar in patients with and without HFpEF (both OR = 1.11; *p* for interaction = 0.607). A significant interaction was observed for CKD status (*p* for interaction = 0.018). The association between UAR and in-hospital mortality remained significant in patients without CKD (OR = 1.13, 95% CI: 1.07 to 1.20), whereas it was attenuated and not statistically significant in patients with CKD (OR = 1.04, 95% CI: 0.96 to 1.12).

For LVEF and RVID, UAR was consistently associated with lower LVEF and greater RVID across all analyzed subgroups. Several significant interactions were observed, particularly for sex and CKD. The inverse association between UAR and LVEF was stronger in males than in females (β = −0.64, 95% CI: −0.73 to −0.55 vs. β = −0.36, 95% CI: −0.43 to −0.30; *p* for interaction < 0.001), and the positive association between UAR and RVID was also stronger in males than in females (β = 0.24, 95% CI: 0.20 to 0.29 vs. β = 0.18, 95% CI: 0.13 to 0.22; *p* for interaction = 0.009). In addition, the associations of UAR with both LVEF and RVID were attenuated in patients with CKD compared with those without CKD. For LVEF, the β coefficients were −0.34 (95% CI: −0.48 to −0.21) in patients with CKD and −0.50 (95% CI: −0.56 to −0.43) in those without CKD (*p* for interaction < 0.001). For RVID, the β coefficients were 0.13 (95% CI: 0.07 to 0.20) and 0.24 (95% CI: 0.20 to 0.28), respectively (*p* for interaction = 0.007). For clinically recorded PH severity grades, the association between UAR and LVEF differed across mild, moderate, and severe PH (*p* for interaction < 0.001), whereas no significant interaction was observed for RVID (*p* for interaction = 0.910).

### 3.6. The Predictive Value and Incremental Performance of UAR for In-Hospital Mortality in PH

**Figure 4:**
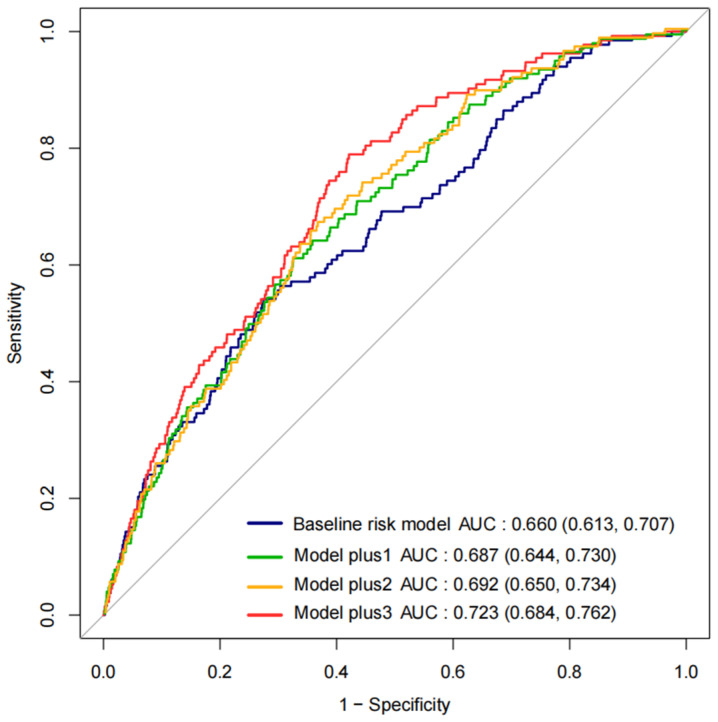
ROC curves comparing prediction models for in-hospital mortality. Model construction was the same as described in Table 4. AUC, area under the curve; CI, confidence interval.

Receiver operating characteristic (ROC) analysis was employed to assess the predictive performance of different models for in-hospital mortality in patients with PH. As shown in Figure 4 and Table 4, the baseline risk model, which included demographic characteristics, comorbidities, and clinically recorded PH severity grade, yielded a C-statistic of 0.6601 (95% CI: 0.6133 to 0.7069). After adding routinely available laboratory variables, including NT-proBNP, creatinine, lipid profiles, liver function markers, and PaO_2_, the C-statistic increased to 0.6872 (95% CI: 0.6444 to 0.7300). Further addition of echocardiographic parameters, including LVEF, LVEDD, RVID, and RAID, resulted in a C-statistic of 0.6922 (95% CI: 0.6503 to 0.7342). Notably, when UAR was further incorporated into this clinical-laboratory-echocardiographic model, the C-statistic increased to 0.7230 (95% CI: 0.6837 to 0.7624).

The incremental predictive value of UAR was further supported by reclassification analyses. Compared with the model including clinical, laboratory, and echocardiographic variables, the addition of UAR significantly improved model discrimination and reclassification, with an increase in the C-statistic (*p* = 0.006), an NRI of 36.69% (95% CI: 19.71% to 53.67%, *p* < 0.0001), and an IDI of 0.19% (95% CI: 0.01% to 0.37%, *p* = 0.0396). These findings suggest that UAR may provide additional prognostic information for in-hospital mortality beyond routine variables in patients with PH.

## 4. Discussion

Most of the existing research on the mechanisms linking oxidative stress and pulmonary hypertension (PH) has been conducted at the in vitro experimental level. In contrast, studies exploring the association between oxidative stress and in-hospital risk stratification in PH patients are relatively scarce [29]. To the best of our knowledge, this is the first study to systematically evaluate the clinical relevance of UAR for short-term in-hospital risk stratification in hospitalized patients with clinically diagnosed PH, including its associations with in-hospital mortality, clinically recorded PH severity grades, and selected cardiac structural and functional indicators. In this retrospective cohort, higher UAR was independently associated with increased odds of in-hospital mortality and greater odds of being classified into a higher clinically recorded PH severity grade. Higher UAR was also associated with lower LVEF and greater RVID, suggesting its relationship with poorer cardiac functional status, more prominent cardiac remodeling, and greater overall cardiac burden. In addition, adding UAR to a model containing routinely available clinical, laboratory, and echocardiographic variables improved discrimination and reclassification for in-hospital mortality. These findings support the potential role of UAR as a complementary admission biomarker for short-term in-hospital risk stratification in patients with PH.

Previous studies have shown that serum uric acid (UA) and albumin (ALB) are associated with mortality and adverse outcomes in patients with PH [13,14,15]. However, either marker alone may provide incomplete information. UA has a dual role in redox homeostasis: elevated UA may promote oxidative stress and inflammation under conditions such as hypoxia, ischemia, chronic inflammation, and endothelial dysfunction, whereas UA may also act as an endogenous antioxidant in certain contexts [10,21,30,31,32,33,34,35]. ALB reflects nutritional and inflammatory status and also contributes to antioxidant defense; lower ALB levels have been linked to adverse cardiovascular outcomes [36,37]. Therefore, UAR, as a composite biomarker integrating UA and ALB, may better reflect the combined burden of oxidative stress, inflammation, nutritional impairment, and renal-metabolic dysfunction. This biological rationale may partly explain the observed associations of higher UAR with in-hospital mortality, clinically recorded PH severity grades, lower LVEF, and greater RVID in hospitalized patients with PH.

The nonlinear associations observed in the present study suggest that UAR should be interpreted as an integrated risk-burden marker rather than a purely cardiac-specific indicator. For in-hospital mortality and LVEF, the attenuation of associations above the identified thresholds may reflect a plateau phenomenon and increasing clinical heterogeneity among patients with very high UAR, rather than a true reduction in risk. At lower-to-moderate UAR levels, increases in UAR may more directly reflect worsening cardiopulmonary stress, oxidative injury, inflammation, and nutritional impairment. However, at very high UAR levels, the ratio may be increasingly influenced by overlapping systemic conditions, including renal dysfunction, marked hypoalbuminemia, systemic inflammation, malnutrition, and multiorgan stress. In this setting, further increases in UAR may no longer translate into a proportional increase in mortality risk or a proportional decrease in LVEF, because these patients may already represent a high-risk and biologically heterogeneous subgroup [38]. The subgroup analyses further support this interpretation and help clarify potential effect modification. The broadly consistent association between UAR and in-hospital mortality across most clinically relevant subgroups, including patients with and without HFpEF, suggests that the prognostic relevance of UAR is not confined to a single LVEF phenotype. In contrast, the attenuated association in patients with CKD suggests that: in patients with CKD, elevated UAR may be driven more strongly by impaired uric acid excretion and renal-metabolic burden, thereby reducing its specificity for cardiopulmonary or cardiac remodeling-related risk. The attenuated association in the severe PH subgroup should be interpreted cautiously because this subgroup had fewer mortality events and greater clinical heterogeneity; advanced PH-related mortality may be more strongly driven by overt right-heart failure, systemic congestion, and multiorgan decompensation than by incremental changes in UAR. The stronger associations of UAR with LVEF and RVID in males may also have biological plausibility. Previous studies have reported sex-related differences in right ventricular adaptation and prognosis in pulmonary hypertension, with right ventricular function contributing to sex differences in survival [39].

Current PH evaluation emphasizes the integration of clinical status, biomarkers, imaging findings, and hemodynamic information rather than reliance on a single indicator [7]. Therefore, the clinical relevance of UAR lies not in replacing established markers, but in whether it captures residual risk information that is not fully represented by routinely available variables. In hospitalized patients with PH, short-term mortality may reflect not only cardiopulmonary impairment, but also systemic vulnerability related to renal dysfunction, hepatic congestion, inflammation, oxidative stress, malnutrition, and multiorgan stress. UAR may integrate several of these dimensions through the combined information provided by uric acid and albumin, which may partly explain why it improved risk discrimination and reclassification when added to clinical, laboratory, and echocardiographic models. Previous methodological work has emphasized that the value of a new marker should be assessed by whether it improves risk classification beyond existing models, rather than by statistical association alone [40]. From this perspective, our findings suggest that UAR may serve as a practical admission-based complementary marker to refine short-term in-hospital risk stratification in patients with PH, particularly when complete functional or hemodynamic assessment is not readily available.

Multiple studies have demonstrated that UAR is associated with disease severity and prognosis across various cardiovascular conditions. In patients with heart failure, Liu et al. reported that elevated UAR was associated with a higher risk of short-term death or hospital readmission [17]. In coronary artery disease (CAD), UAR has been linked to mortality and new-onset atrial fibrillation in ST-elevation myocardial infarction, higher SYNTAX scores in non-ST-segment elevation myocardial infarction, and chronic CAD severity [18,22,41,42]. UAR has also been associated with adverse outcomes in several interventional or high-risk cardiovascular settings, including major adverse cardiac and cerebral events after transcatheter aortic valve implantation, long-term mortality in acute type A aortic dissection, in-stent restenosis after drug-eluting stent implantation, and long-term cardiac mortality after percutaneous coronary intervention for unstable angina pectoris [19,20,43,44]. Beyond cardiovascular diseases, UAR has also been reported as a prognostic marker for all-cause and cardiovascular mortality in patients with diabetes [44]. Taken together, these findings indicate that UAR may represent a broadly informative risk marker across cardiometabolic diseases. This accumulated evidence supports the scientific rationale for investigating UAR in PH and provides external consistency for the associations observed in the present study.

Because this study focused on hospitalized patients with clinically diagnosed PH and in-hospital mortality, our results do not directly establish the role of UAR in PH screening, definitive diagnosis, etiologic classification, guideline-based risk assessment, or long-term prognosis prediction. Rather, the current evidence supports the potential use of UAR as an early complementary marker for short-term in-hospital risk stratification. This clinical positioning is supported by several practical advantages of UAR: it is inexpensive, can be readily derived from routinely available admission laboratory tests, and integrates information related to oxidative stress, inflammation, nutritional status, and renal-metabolic burden. In this setting, elevated UAR may provide additional information beyond conventional clinical, laboratory, and imaging markers, helping clinicians identify hospitalized PH patients with a higher short-term risk profile who may require closer monitoring and more comprehensive evaluation.

This study has several limitations. First, as a single-center retrospective study based on real-world electronic medical records, residual confounding and information bias could not be completely avoided. Although PH identification was guided by current recommendations and right-heart catheterization data were considered when available, complete hemodynamic data were not available for all patients; therefore, this study focused on hospitalized patients with clinically diagnosed PH rather than a uniformly right-heart catheterization-confirmed cohort. Similarly, several clinically important variables, including WHO functional class, 6 min walk distance, detailed right ventricular functional parameters, critical-care variables, and urate-lowering therapy, were not systematically available. Second, because complete etiologic work-up was not consistently available over the long study period, we could not reliably classify all patients according to ESC/ERS PH groups. Clinically recorded PH severity grades and available clinical subgroups were therefore used to partially address disease heterogeneity. Therefore, UAR should be interpreted as a complementary admission-based marker for short-term in-hospital risk stratification. Future larger prospective multicenter studies with comprehensive PH phenotyping, standardized risk assessment, and long-term follow-up are needed to validate these findings and clarify the clinical utility of UAR. Further research is also needed to determine whether UAR-guided risk stratification can improve clinical decision-making or patient outcomes.

## 5. Conclusions

Higher UAR was independently associated with increased in-hospital mortality, higher clinically recorded PH severity grades, lower LVEF, and greater RVID. Adding UAR to models incorporating routinely available clinical, laboratory, and echocardiographic variables improved the prediction of in-hospital mortality, suggesting that UAR may provide complementary prognostic information beyond conventional markers. Given its low cost, routine availability at admission, and ability to integrate information related to oxidative stress, inflammation, nutritional status, and renal-metabolic burden, UAR may serve as a practical complementary biomarker for short-term in-hospital risk stratification in patients with PH.

## Figures and Tables

**Table 1:** Baseline characteristics according to UAR quartiles.

Variables	Total (n = 8763)	1.73 ≤ Q1 < 6.49 (n = 2200)	6.49 ≤ Q2 < 8.33 (n = 2172)	8.33 ≤ Q3 < 10.88 (n = 2192)	10.88 ≤ Q4 < 25.01 (n = 2199)	*p*-Value
Demographics						
Age (years)	64.6 ± 15.0	63.7 ± 14.9	65.6 ± 14.2	66.2 ± 14.5	63.0 ± 16.1	<0.001
Sex, n (%)						<0.001
Male	3973 (45.3)	543 (24.7)	887 (40.8)	1179 (53.8)	1364 (62)	
Female	4790 (54.7)	1657 (75.3)	1285 (59.2)	1013 (46.2)	835 (38)	
Race, n (%)						<0.001
Han ethnicity	5062 (57.8)	1185 (53.9)	1225 (56.4)	1298 (59.2)	1354 (61.6)	
Other ethnicity	3701 (42.2)	1015 (46.1)	947 (43.6)	894 (40.8)	845 (38.4)	
BMI (kg/m^2^)	24.9 ± 10.2	23.9 ± 5.8	25.2 ± 15.7	25.0 ± 7.5	25.4 ± 8.7	<0.001
Comorbidities						
Hypertension, n (%)						<0.001
No	4699 (53.6)	1315 (59.8)	1195 (55)	1119 (51)	1070 (48.7)	
Yes	4064 (46.4)	885 (40.2)	977 (45)	1073 (49)	1129 (51.3)	
Diabetes, n (%)						<0.001
No	7241 (82.6)	1857 (84.4)	1849 (85.1)	1808 (82.5)	1727 (78.5)	
Yes	1522 (17.4)	343 (15.6)	323 (14.9)	384 (17.5)	472 (21.5)	
CKD, n (%)						<0.001
No	7530 (85.9)	2125 (96.6)	2036 (93.7)	1929 (88)	1440 (65.5)	
Yes	1233 (14.1)	75 (3.4)	136 (6.3)	263 (12)	759 (34.5)	
CTD, n (%)						0.115
No	8654 (98.8)	2183 (99.2)	2144 (98.7)	2162 (98.6)	2165 (98.5)	
Yes	109 (1.2)	17 (0.8)	28 (1.3)	30 (1.4)	34 (1.5)	
PH Severity, n (%)						<0.001
Mild	4738 (54.1)	1440 (65.5)	1302 (59.9)	1170 (53.4)	826 (37.6)	
Moderate	2782 (31.7)	578 (26.3)	623 (28.7)	674 (30.7)	907 (41.2)	
Severe	1243 (14.2)	182 (8.3)	247 (11.4)	348 (15.9)	466 (21.2)	
Vital signs						
SBP (mmHg)	127.0 ± 20.4	126.9 ± 19.4	127.9 ± 19.8	127.1 ± 20.0	126.3 ± 22.2	0.077
DBP (mmHg)	75.6 ± 14.6	74.8 ± 11.6	75.6 ± 12.1	75.6 ± 13.4	76.2 ± 19.8	0.024
HR (beats/min)	82.7 ± 17.7	80.7 ± 13.5	81.4 ± 14.1	82.7 ± 23.8	86.1 ± 17.0	<0.001
Echocardiography						
LVEF (%)	58.6 ± 9.0	61.4 ± 5.8	60.2 ± 7.4	58.5 ± 8.8	54.4 ± 11.5	<0.001
LVEF-defined phenotype, n (%)						<0.001
≤40	692 (7.9)	46 (2.1)	101 (4.7)	167 (7.6)	378 (17.2)	
41–49	602 (6.9)	74 (3.4)	93 (4.3)	156 (7.1)	279 (12.7)	
≥50	7469 (85.2)	2080 (94.5)	1978 (91.1)	1869 (85.3)	1542 (70.1)	
LAID (mm)	39.2 ± 7.7	36.7 ± 6.5	38.3 ± 7.0	40.1 ± 8.0	41.9 ± 8.2	<0.001
LVESD (mm)	34.6 ± 8.3	32.0 ± 5.4	33.4 ± 6.8	34.8 ± 8.0	38.4 ± 10.7	<0.001
LVEDD (mm)	50.0 ± 8.0	47.6 ± 5.6	49.0 ± 6.7	50.3 ± 7.7	53.2 ± 10.2	<0.001
RAID (mm)	38.5 ± 7.3	36.5 ± 6.0	37.6 ± 6.3	38.8 ± 7.3	41.1 ± 8.3	<0.001
RVID (mm)	21.0 ± 5.0	20.3 ± 5.6	20.5 ± 4.2	21.1 ± 4.5	22.2 ± 5.5	<0.001
RVOT (mm)	30.5 ± 5.3	29.5 ± 5.0	29.9 ± 4.9	30.6 ± 5.2	31.9 ± 5.9	<0.001
Laboratory data						
NT-ProBNP (pg/mL)	583.0 (159.0, 2400.0)	322.0 (114.0, 1200.0)	390.0 (123.8, 1560.0)	630.5 (169.0, 2355.0)	1820.0 (393.5, 5330.0)	<0.001
WBC (109/L)	6.4 (5.1, 8.0)	6.1 (4.8, 7.6)	6.2 (5.0, 7.7)	6.4 (5.2, 8.0)	6.8 (5.3, 8.7)	<0.001
Lymphocyte (109/L)	1.5 (1.1, 2.0)	1.6 (1.1, 2.0)	1.6 (1.1, 2.1)	1.5 (1.1, 2.0)	1.3 (0.9, 1.8)	<0.001
Monocyte (109/L)	0.5 (0.4, 0.7)	0.5 (0.4, 0.6)	0.5 (0.4, 0.6)	0.5 (0.4, 0.7)	0.6 (0.4, 0.7)	<0.001
Neutrophil (109/L)	3.9 (2.9, 5.4)	3.6 (2.7, 5.0)	3.6 (2.8, 5.0)	4.0 (3.0, 5.4)	4.6 (3.3, 6.3)	<0.001
Hb (g/L)	126.2 ± 25.8	124.9 ± 20.4	128.2 ± 22.2	129.2 ± 25.3	122.4 ± 32.8	<0.001
PLT (109/L)	218.3 ± 87.1	229.1 ± 83.1	222.1 ± 84.1	215.5 ± 84.0	206.6 ± 94.9	<0.001
ALT (U/L)	20.3 (14.5, 30.7)	19.0 (14.0, 27.6)	20.0 (14.3, 28.9)	20.8 (15.0, 30.1)	22.1 (15.0, 38.0)	<0.001
AST (U/L)	26.2 (20.4, 34.9)	25.5 (19.7, 33.1)	25.1 (19.7, 32.6)	26.3 (20.4, 34.4)	28.7 (21.9, 40.8)	<0.001
ALB (g/L)	37.3 ± 5.5	39.0 ± 5.1	38.6 ± 4.9	37.5 ± 5.0	34.3 ± 5.5	<0.001
GLO (g/L)	30.8 ± 6.6	30.6 ± 6.2	30.4 ± 6.3	31.0 ± 6.8	31.3 ± 7.1	<0.001
Creatinine (μmol/L)	68.8 (56.1, 86.9)	56.6 (48.1, 67.0)	64.6 (55.3, 77.7)	72.4 (60.8, 88.1)	91.2 (71.9, 127.0)	<0.001
Urea (mmol/L)	6.2 (4.9, 8.0)	5.2 (4.2, 6.4)	5.8 (4.7, 7.1)	6.4 (5.1, 8.0)	8.2 (6.1, 12.4)	<0.001
UA (μmol/L)	332.2 ± 121.3	206.8 ± 46.2	285.3 ± 41.3	354.1 ± 53.1	482.2 ± 105.6	<0.001
Glucose (mmol/L)	6.0 ± 2.6	6.0 ± 2.7	5.8 ± 2.1	5.9 ± 2.3	6.3 ± 3.0	<0.001
TC (mmol/L)	3.7 ± 1.0	3.8 ± 1.0	3.8 ± 1.0	3.7 ± 1.0	3.5 ± 1.1	<0.001
TG (mmol/L)	1.3 ± 0.8	1.3 ± 0.7	1.3 ± 0.7	1.4 ± 1.0	1.3 ± 0.8	<0.001
HDL-C (mmol/L)	1.0 ± 0.3	1.1 ± 0.4	1.0 ± 0.3	1.0 ± 0.3	0.9 ± 0.3	<0.001
LDL-C (mmol/L)	2.3 ± 0.8	2.3 ± 0.8	2.3 ± 0.8	2.3 ± 0.8	2.2 ± 0.9	<0.001
pH	7.4 ± 0.1	7.4 ± 0.0	7.4 ± 0.0	7.4 ± 0.1	7.4 ± 0.1	0.002
CaO_2_ (mL/dL)	19.4 ± 4.4	19.4 ± 4.1	19.6 ± 4.1	19.8 ± 4.4	18.9 ± 4.9	<0.001
PaO_2_ (mmHg)	107.0 (81.3, 336.9)	105.0 (82.1, 339.5)	107.0 (82.4, 342.9)	107.0 (81.3, 336.7)	108.0 (79.3, 323.8)	0.452
SaO_2_ (%)	93.9 ± 6.1	94.4 ± 5.2	94.1 ± 5.5	93.7 ± 6.5	93.4 ± 7.1	<0.001
UAR	9.1 ± 3.7	5.3 ± 0.9	7.4 ± 0.5	9.5 ± 0.7	14.2 ± 3.0	<0.001
Outcome						
Death, n (%)						<0.001
No	8630 (98.5)	2188 (99.5)	2151 (99)	2153 (98.2)	2138 (97.2)	
Yes	133 (1.5)	12 (0.5)	21 (1)	39 (1.8)	61 (2.8)	

BMI, body mass index; CKD, chronic kidney disease; CTD, connective tissue disease; PH, pulmonary hypertension; SBP, systolic blood pressure; DBP, diastolic blood pressure; HR, heart rate; LAID, left atrial internal dimension; LVESD, left ventricular end-systolic dimension; LVEDD, left ventricular end-diastolic dimension; LVEF, left ventricular ejection fraction; RAID, right atrial internal dimension; RVID, right ventricular internal dimension; RVOT, right ventricular outflow tract; NT-ProBNP, N-terminal pro-B-type natriuretic peptide; WBC, white blood cell count; Hb, hemoglobin; PLT, platelet count; ALT, alanine aminotransferase; AST, aspartate aminotransferase; ALB, albumin; GLO, globulin; UA, uric acid; TC, total cholesterol; TG, triglycerides; HDL-C, high-density lipoprotein cholesterol; LDL-C, low-density lipoprotein cholesterol; pH, potential of hydrogen; CaO_2_, arterial oxygen content; PaO_2_, partial pressure of arterial oxygen; SaO_2_, arterial oxygen saturation; (blood gas variables, including PaO_2_, SaO_2_, and CaO_2_, were obtained from routine clinical blood gas testing during hospitalization and were not restricted to measurements under room-air conditions); UAR, uric acid to albumin ratio.

**Table 2:** Association of UAR with clinically recorded PH severity grades in ordinal logistic regression.

Variables	UAR (n = 8763)		UAR Levels Quartiles						
			Q1 (n = 2200)	Q2 (n = 2172)		Q3 (n = 2192)		Q4 (n = 2199)	
	OR (95%CI)	*p*_Value	OR (95%CI)	OR (95%CI)	*p*_Value	OR (95%CI)	*p*_Value	OR (95%CI)	*p*_Value
Unadjusted	1.11 (1.08~1.13)	<0.001	1 (Ref)	1.28 (1.14~1.45)	<0.001	1.73 (1.54~1.95)	<0.001	3.02 (2.69~3.41)	<0.001
Model 1	1.12 (1.11~1.13)	<0.001	1 (Ref)	1.34 (1.18~1.51)	<0.001	1.84 (1.63~2.08)	<0.001	3.17 (2.81~3.58)	<0.001
Model 2	1.13 (1.11~1.14)	<0.001	1 (Ref)	1.34 (1.19~1.52)	<0.001	1.86 (1.65~2.11)	<0.001	3.27 (2.88~3.72)	<0.001
Model 3	1.12 (1.10~1.14)	<0.001	1 (Ref)	1.33 (1.22~1.45)	<0.001	1.84 (1.71~1.99)	<0.001	3.11 (2.92~3.32)	<0.001
Model 4	1.11 (1.09~1.13)	<0.001	1 (Ref)	1.33 (1.22~1.45)	<0.001	1.82 (1.69~1.96)	<0.001	2.99 (2.81~3.19)	<0.001

Model 1: Adjusted for Age + Sex + Race + BMI. Model 2: Adjusted for model 1 + Hypertension + Diabetes + CKD + CTD. Model 3: Adjusted for model 2 + NT_ProBNP + Creatinine + TC + TG + HDL + LDL. Model 4: Adjusted for model 3 + CaO_2_ + SaO_2_ + PaO_2_. OR, odds ratio; 95% CI, 95% confidence interval.

**Table 3:** Associations of UAR with in-hospital mortality, LVEF, and RVID by multivariable regression analysis.

Variables	UAR (n = 8763)		UAR Levels Quartiles						
			Q1 (n = 2200)	Q2 (n = 2172)		Q3 (n = 2192)		Q4 (n = 2199)	
In-hospital mortality									
	OR (95%CI)	*p*_value	OR (95%CI)	OR (95%CI)	*p*_value	OR (95%CI)	*p*_value	OR (95%CI)	*p*_value
Unadjusted	1.13 (1.09~1.18)	<0.001	1 (Ref)	1.78 (0.87~3.63)	0.112	3.30 (1.73~6.32)	0.001	5.20 (2.79~9.69)	<0.001
Model 1	1.13 (1.09~1.18)	<0.001	1 (Ref)	1.77 (0.86~3.61)	0.119	3.21 (1.66~6.2)	0.001	5.14 (2.72~9.71)	<0.001
Model 2	1.12 (1.07~1.17)	<0.001	1 (Ref)	1.75 (0.85~3.57)	0.126	3.10 (1.60~6.00)	0.001	4.50 (2.33~8.68)	<0.001
Model 3	1.11 (1.07~1.16)	<0.001	1 (Ref)	1.75 (0.86~3.59)	0.125	3.09 (1.59~6.01)	0.001	4.38 (2.25~8.53)	<0.001
Model 4	1.09 (1.04~1.14)	<0.001	1 (Ref)	1.74 (0.85~3.57)	0.128	3.06 (1.57~5.95)	0.001	4.28 (2.2~8.35)	<0.001
LVEF									
	β (95%CI)	*p*_value	β (95%CI)	β (95%CI)	*p*_value	β (95%CI)	*p*_value	β (95%CI)	*p*_value
Unadjusted	−0.73 (−0.78~−0.68)	<0.001	1 (Ref)	−1.24 (−1.76~−0.73)	<0.001	−2.9 (−3.41~−2.39)	<0.001	−6.96 (−7.47~−6.45)	<0.001
Model 1	−0.62 (−0.67~−0.57)	<0.001	1 (Ref)	−0.8 (−1.31~−0.29)	0.002	−2.1 (−2.61~−1.58)	<0.001	−5.82 (−6.34~−5.3)	<0.001
Model 2	−0.57 (−0.63~−0.52)	<0.001	1 (Ref)	−0.82 (−1.32~−0.31)	0.002	−2.01 (−2.53~−1.49)	<0.001	−5.35 (−5.9~−4.8)	<0.001
Model 3	−0.52 (−0.57~−0.46)	<0.001	1 (Ref)	−0.75 (−1.26~−0.25)	0.004	−1.89 (−2.41~−1.37)	<0.001	−4.85 (−5.4~−4.29)	<0.001
Model 4	−0.53 (−0.58~−0.47)	<0.001	1 (Ref)	−0.77 (−1.27~−0.26)	0.003	−1.94 (−2.45~−1.42)	<0.001	−4.95 (−5.5~−4.39)	<0.001
RVID									
	β (95%CI)	*p*_value	β (95%CI)	β (95%CI)	*p*_value	β (95%CI)	*p*_value	β (95%CI)	*p*_value
Unadjusted	0.21 (0.18~0.24)	<0.001	1 (Ref)	0.19 (−0.10~0.49)	0.195	0.76 (0.47~1.06)	<0.001	1.87 (1.57~2.16)	<0.001
Model 1	0.17 (0.14~0.20)	<0.001	1 (Ref)	0.18 (−0.11~0.47)	0.215	0.68 (0.39~0.97)	<0.001	1.50 (1.20~1.79)	<0.001
Model 2	0.20 (0.17~0.23)	<0.001	1 (Ref)	0.21 (−0.07~0.50)	0.143	0.80 (0.50~1.09)	<0.001	1.89 (1.58~2.20)	<0.001
Model 3	0.22 (0.19~0.25)	<0.001	1 (Ref)	0.23 (−0.06~0.52)	0.114	0.82 (0.52~1.11)	<0.001	1.88 (1.57~2.20)	<0.001
Model 4	0.18 (0.15~0.22)	<0.001	1 (Ref)	0.22 (−0.07~0.50)	0.14	0.76 (0.46~1.05)	<0.001	1.79 (1.48~2.11)	<0.001

Model 1: Adjusted for Age + Sex + Race + BMI. Model 2: Adjusted for model 1 + Hypertension + Diabetes + CKD + CTD. Model 3: Adjusted for model 2 + NT-proBNP, creatinine, TC, TG, HDL-C, and LDL-C. Model 4: Adjusted for model 3 + CaO_2_, SaO_2_, and PaO_2_. OR, odds ratio; 95% CI, 95% confidence interval. OR was reported for in-hospital mortality, and β coefficients were reported for LVEF and RVID.

**Table 4:** Incremental predictive value of various models and UAR with C-statistics, NRI, and IDI.

Model	C-Statistic (95%CI)	*p*-Value	NRI (95%CI)	*p*-Value	IDI (95%CI)	*p*-Value
Various model incremental predictive value						
Baseline risk model	0.6601 (0.6133~0.7069)		Ref		Ref	
Model plus1	0.6872 (0.6444~0.7300)	0.011	14.04% (−3.03%–31.11%)	0.1069	0.15% (0.02%–0.28%)	0.0276
Model plus2	0.6922 (0.6503~0.7342)	0.005	30.19% (13.32%–47.06%)	0.0005	0.18% (0.04%–0.33%)	0.0134
Model plus3	0.7230 (0.6837~0.7624)	<0.001	49.49% (32.84%–66.15%)	<0.0001	0.37% (0.13%–0.61%)	0.0027
Incremental predictive value of UAR						
Model plus2	0.6922 (0.6503~0.7342)		Ref		Ref	
Model plus2 + UAR	0.7230 (0.6837~0.7624)	0.006	36.69% (19.71%–53.67%)	<0.0001	0.19% (0.01%–0.37%)	0.0396

Baseline risk model: Age + Sex + Race + BMI + Hypertension + Diabetes + CKD + CTD + PH Severity. Model plus1: Baseline risk model + NT-proBNP + creatinine + TC + TG + HDL-C + LDL-C + ALT + AST + PaO_2_. Model plus2: Model plus1 + LVEF + LVEDD + RVID + RAID. Model plus3: Model plus2 + UAR. NRI, net reclassification improvement; IDI, integrated discrimination improvement; CI, confidence interval.

## Data Availability

The data are not publicly available because they contain potentially identifiable clinical information, but are available from the first author upon reasonable request.

## Supplementary Materials

The following supporting information can be downloaded at: https://www.mdpi.com/article/10.3390/jcdd13070295/s1, Table S1. Baseline characteristics according to clinically recorded PH severity grades; Table S2. Factors associated with in-hospital mortality of PH; Table S3. Threshold effect analyses for the associations of UAR with in-hospital mortality, LVEF, and RVID.
