# Supplementary material for: Uric Acid-to-Albumin Ratio as a Complementary Biomarker for In-Hospital Risk Stratification in Patients with Pulmonary Hypertension: A Retrospective Cohort Study
Source: J Cardiovasc Dev Dis. 2026 Jun 25;13(7):295. doi: 10.3390/jcdd13070295 (PMC13410907; doi:10.3390/jcdd13070295)
Supplement: Supplementary file 1 [file jcdd-13-00295-s001.zip › jcdd-4285605-supplementary.pdf]

**Table S1. Baseline characteristics according to clinically recorded PH severity grades**

| Variables | Total (n = 8763) | Mild PH (n = 4738) | Moderate PH (n = 2782) | Severe PH (n = 1243) | P-value |
|---|---|---|---|---|---|
| **Demographics** | | | | | |
| Age(years) | 64.6 ± 15.0 | 65.7 ± 14.3 | 64.0 ± 15.6 | 62.0 ± 16.0 | < 0.001 |
| Sex,n(%) | | | | | < 0.001 |
| Male | 3973 (45.3) | 2020 (42.6) | 1374 (49.4) | 579 (46.6) | |
| Female | 4790 (54.7) | 2718 (57.4) | 1408 (50.6) | 664 (53.4) | |
| Race,n(%) | | | | | < 0.001 |
| Han ethnicity | 5062 (57.8) | 2883 (60.8) | 1540 (55.4) | 639 (51.4) | |
| Other ethnicity | 3701 (42.2) | 1855 (39.2) | 1242 (44.6) | 604 (48.6) | |
| BMI(kg/m2) | 24.9 ± 10.2 | 25.0 ± 10.5 | 24.9 ± 9.2 | 24.6 ± 10.9 | 0.48 |
| **Comorbidities** | | | | | |
| Hypertension,n(%) | | | | | < 0.001 |
| No | 4699 (53.6) | 2477 (52.3) | 1469 (52.8) | 753 (60.6) | |
| Yes | 4064 (46.4) | 2261 (47.7) | 1313 (47.2) | 490 (39.4) | |
| Diabetes,n(%) | | | | | 0.003 |
| No | 7241 (82.6) | 3928 (82.9) | 2253 (81) | 1060 (85.3) | |
| Yes | 1522 (17.4) | 810 (17.1) | 529 (19) | 183 (14.7) | |
| CKD,n(%) | | | | | < 0.001 |
| No | 7530 (85.9) | 4189 (88.4) | 2270 (81.6) | 1071 (86.2) | |
| Yes | 1233 (14.1) | 549 (11.6) | 512 (18.4) | 172 (13.8) | |
| CTD,n(%) | | | | | < 0.001 |
| No | 8654 (98.8) | 4685 (98.9) | 2756 (99.1) | 1213 (97.6) | |
| Yes | 109 ( 1.2) | 53 (1.1) | 26 (0.9) | 30 (2.4) | |
| **Vital signs** | | | | | |
| SBP(mmHg) | 127.0 ± 20.4 | 127.5 ± 19.6 | 127.4 ± 21.4 | 124.7 ± 21.0 | < 0.001 |
| DBP(mmHg) | 75.6 ± 14.6 | 75.1 ± 12.2 | 76.3 ± 18.3 | 75.6 ± 13.6 | 0.003 |
| HR(beats/min) | 82.7 ± 17.7 | 80.9 ± 19.1 | 84.4 ± 16.0 | 86.1 ± 15.1 | < 0.001 |
| **Echocardiography** | | | | | |
| LVEF(%) | 58.6 ± 9.0 | 59.9 ± 7.6 | 56.2 ± 10.7 | 59.0 ± 9.1 | < 0.001 |
| LVEF-defined phenotype,n(%) | | | | | < 0.001 |
| ≤40 | 692 ( 7.9) | 240 (5.1) | 357 (12.8) | 95 (7.6) | |
| 41-49 | 602 ( 6.9) | 248 (5.2) | 281 (10.1) | 73 (5.9) | |
| ≥50 | 7469 (85.2) | 4250 (89.7) | 2144 (77.1) | 1075 (86.5) | |
| LAID(mm) | 39.2 ± 7.7 | 38.1 ± 6.5 | 41.2 ± 8.4 | 39.2 ± 9.3 | < 0.001 |
| LVESD(mm) | 34.6 ± 8.3 | 33.8 ± 6.8 | 36.8 ± 9.9 | 33.0 ± 8.7 | < 0.001 |
| LVEDD(mm) | 50.0 ± 8.0 | 49.5 ± 6.6 | 51.9 ± 9.4 | 47.7 ± 8.9 | < 0.001 |
| RAID(mm) | 38.5 ± 7.3 | 35.9 ± 5.3 | 40.2 ± 7.5 | 44.7 ± 8.4 | < 0.001 |
| RVID(mm) | 21.0 ± 5.0 | 19.5 ± 3.4 | 21.7 ± 4.8 | 25.2 ± 7.4 | < 0.001 |
| RVOT(mm) | 30.5 ± 5.3 | 28.8 ± 3.8 | 31.4 ± 5.6 | 35.0 ± 6.7 | < 0.001 |
| **Laboratory data** | | | | | |
| NT-ProBNP(pg/mL) | 583.0 (159.0, 2400.0) | 409.0 (129.0, 1740.0) | 862.0 (206.2, 3140.0) | 1030.0 (251.0, 3360.0) | < 0.001 |
| WBC(109/L) | 6.4 (5.1, 8.0) | 6.2 (4.9, 7.8) | 6.4 (5.2, 8.2) | 6.7 (5.4, 8.5) | < 0.001 |
| Lymphocyte(109/L) | 1.5 (1.1, 2.0) | 1.6 (1.1, 2.1) | 1.4 (1.0, 1.9) | 1.3 (0.9, 1.8) | < 0.001 |
| Monocyte(109/L) | 0.5 (0.4, 0.7) | 0.5 (0.4, 0.6) | 0.5 (0.4, 0.7) | 0.5 (0.4, 0.7) | < 0.001 |
| Neutrophil(109/L) | 3.9 (2.9, 5.4) | 3.7 (2.8, 5.1) | 4.1 (3.1, 5.7) | 4.5 (3.3, 6.1) | < 0.001 |

| | | | | | |
|---|---|---|---|---|---|
| Hb(g/L) | 126.2 ± 25.8 | 125.5 ± 23.3 | 125.0 ± 27.4 | 131.2 ± 30.1 | < 0.001 |
| PLT(109/L) | 218.3 ± 87.1 | 220.7 ± 88.3 | 216.3 ± 86.4 | 213.9 ± 83.5 | 0.016 |
| ALT(U/L) | 20.3 (14.5, 30.7) | 20.0 (14.3, 29.0) | 21.0 (14.8, 33.0) | 21.4 (15.0, 33.6) | < 0.001 |
| AST(U/L) | 26.2 (20.4, 34.9) | 25.8 (20.0, 33.7) | 26.3 (20.4, 36.2) | 27.8 (21.7, 37.7) | < 0.001 |
| ALB(g/L) | 37.3 ± 5.5 | 38.1 ± 5.4 | 36.6 ± 5.5 | 36.0 ± 5.3 | < 0.001 |
| GLO(g/L) | 30.8 ± 6.6 | 30.6 ± 6.6 | 31.0 ± 6.5 | 31.4 ± 7.1 | < 0.001 |
| Creatinine( μ mol/L) | 68.8 (56.1, 86.9) | 67.0 (55.1, 83.6) | 71.2 (57.8, 93.4) | 69.3 (57.0, 86.8) | < 0.001 |
| Urea(mmol/L) | 6.2 (4.9, 8.0) | 5.9 (4.7, 7.5) | 6.5 (5.1, 8.8) | 6.3 (5.0, 8.5) | < 0.001 |
| UA( μ mol/L) | 332.2 ± 121.3 | 313.3 ± 109.1 | 348.6 ± 129.2 | 367.5 ± 133.6 | < 0.001 |
| Glucose(mmol/L) | 6.0 ± 2.6 | 5.9 ± 2.5 | 6.1 ± 2.7 | 6.1 ± 2.5 | 0.013 |
| TC(mmol/L) | 3.7 ± 1.0 | 3.8 ± 1.0 | 3.6 ± 1.0 | 3.6 ± 1.0 | < 0.001 |
| TG(mmol/L) | 1.3 ± 0.8 | 1.4 ± 0.9 | 1.3 ± 0.7 | 1.2 ± 0.7 | < 0.001 |
| HDL-C(mmol/L) | 1.0 ± 0.3 | 1.0 ± 0.3 | 1.0 ± 0.3 | 0.9 ± 0.3 | < 0.001 |
| LDL-C(mmol/L) | 2.3 ± 0.8 | 2.3 ± 0.8 | 2.3 ± 0.8 | 2.2 ± 0.8 | < 0.001 |
| pH | 7.4 ± 0.1 | 7.4 ± 0.0 | 7.4 ± 0.1 | 7.4 ± 0.1 | 0.002 |
| CaO2(ml/dl) | 19.4 ± 4.4 | 19.6 ± 4.3 | 19.2 ± 4.5 | 19.2 ± 4.5 | < 0.001 |
| PaO2(mmHg) | 107.0 (81.3, 336.9) | 107.0 (83.0, 347.1) | 107.0 (80.4, 329.9) | 106.0 (75.7, 314.3) | < 0.001 |
| SaO2(%) | 93.9 ± 6.1 | 94.6 ± 5.3 | 93.7 ± 6.2 | 91.7 ± 8.1 | < 0.001 |
| UAR | 9.1 ± 3.7 | 8.4 ± 3.2 | 9.7 ± 3.9 | 10.4 ± 4.1 | < 0.001 |
| UAR quartiles,n(%) | | | | | < 0.001 |
| Q1 | 2200 (25.1) | 1440 (30.4) | 578 (20.8) | 182 (14.6) | |
| Q2 | 2172 (24.8) | 1302 (27.5) | 623 (22.4) | 247 (19.9) | |
| Q3 | 2192 (25.0) | 1170 (24.7) | 674 (24.2) | 348 (28) | |
| Q4 | 2199 (25.1) | 826 (17.4) | 907 (32.6) | 466 (37.5) | |
| **Outcome** | | | | | |
| Death,n(%) | | | | | 0.006 |
| No | 8630 (98.5) | 4681 (98.8) | 2736 (98.3) | 1213 (97.6) | |
| Yes | 133 ( 1.5) | 57 (1.2) | 46 (1.7) | 30 (2.4) | |

BMI, body mass index; CKD, chronic kidney disease; CTD, connective tissue disease; PH, pulmonary hypertension; SBP, systolic blood pressure; DBP, diastolic blood pressure; HR, heart rate; LAID, left atrial internal dimension; LVESD, left ventricular end-systolic dimension; LVEDD, left ventricular end-diastolic dimension; LVEF, left ventricular ejection fraction; RAID, right atrial internal dimension; RVID, right ventricular internal dimension; RVOT, right ventricular outflow tract; NT-ProBNP, N-terminal pro-B-type natriuretic peptide; WBC, white blood cell count; Hb, hemoglobin; PLT, platelet count; ALT, alanine aminotransferase; AST, aspartate aminotransferase; ALB, albumin; GLO, globulin; UA, uric acid; TC, total cholesterol; TG, triglycerides; HDL-C, high-density lipoprotein cholesterol; LDL-C, low-density lipoprotein cholesterol; CaO2, arterial oxygen content; PaO2, partial pressure of arterial oxygen; SaO2, arterial oxygen saturation; (blood gas variables, including $PaO_2$, $SaO_2$, and $CaO_2$, were obtained from routine clinical blood gas testing during hospitalization and were not restricted to measurements under room-air conditions); UAR, uric acid to albumin ratio.

**Table S2.Factors associated with in-hospital mortality of PH**

| Variable | OR_95CI | P_value |
|---|---|---|
| Age | 1.01 (1~1.03) | 0.013 |
| Sex | 0.76 (0.55~1.05) | 0.097 |
| Race | 0.44 (0.3~0.64) | <0.001 |
| BMI | 0.97 (0.93~1) | 0.085 |
| Hypertension | 1.18 (0.86~1.64) | 0.309 |
| Diabetes | 1.37 (0.93~2.03) | 0.111 |
| NT.ProBNP | 1 (1~1) | 0.06 |
| Glucose | 1.05 (0.98~1.11) | 0.147 |
| Lymphocyte | 1 (0.99~1.01) | 0.895 |
| Monocyte | 1.02 (1.01~1.04) | 0.001 |
| Neutrophil | 1.01 (1~1.02) | 0.073 |
| Creatinine | 1 (1~1) | 0.376 |
| UA | 1 (1~1) | <0.001 |
| Urea | 1.04 (1.02~1.06) | <0.001 |
| ALT | 1 (1~1) | 0.337 |
| AST | 1 (1~1) | 0.696 |
| ALB | 0.93 (0.9~0.95) | <0.001 |
| TC | 0.82 (0.7~0.97) | 0.024 |
| TG | 0.83 (0.65~1.06) | 0.144 |
| HDL-C | 0.64 (0.39~1.07) | 0.088 |
| LDL-C | 0.84 (0.68~1.03) | 0.091 |
| CaO2 | 0.95 (0.92~0.99) | 0.01 |
| SaO2 | 0.98 (0.96~1) | 0.068 |
| PaO2 | 1 (1~1) | 0.67 |
| UAR | 1.13 (1.09~1.17) | <0.001 |

**Table S3. Threshold effect analyses for the associations of UAR with in-hospital mortality, LVEF, and RVID**

| **In-hospital mortality** | | | |
|---|---|---|---|
| | Threshold of UAR | OR(95%CI) | P-value |
| | <11.3 | 1.298 (1.133~1.486) | <0.001 |
| | ≥11.3 | 1.034 (0.933~1.146) | 0.519 |
| | Likelihood Ratio test | - | 0.002 |
| **LVEF** | | | |
| | Threshold of UAR | Beta(95%CI) | P-value |
| | <16.3 | -0.645(-0.714~-0.576) | <0.001 |
| | ≥16.3 | 0.052(-0.619~0.724) | 0.878 |
| | Likelihood Ratio test | - | 0.001 |
| **RVID** | | | |
| | Threshold of UAR | Beta(95%CI) | P-value |
| | <16.9 | 0.232 (0.193~0.271) | <0.001 |
| | ≥16.9 | 0.483 (0.108~0.858) | 0.012 |
| | Likelihood Ratio test | - | 0.538 |

UAR, uric acid to albumin ratio; OR, odds ratio; 95% CI, 95% confidence interval.
